# A Fast and Label-Free Potentiometric Method for Direct Detection of Glutamine with Silicon Nanowire Biosensors

**DOI:** 10.3390/bios12060368

**Published:** 2022-05-27

**Authors:** Yonghao Jia, Jianyu Wang, Shari Yosinski, Yuehang Xu, Mark A. Reed

**Affiliations:** 1 School of Electronic Science and Engineering, University of Electronic Science and Technology of China, Chengdu 611731, China; yonghao.jia@nwpu.edu.cn; 2 Department of Electrical Engineering, Yale University, New Haven, CT 06511, USA; jywang@ncu.edu.cn (J.W.); shari.yosinski@yale.edu (S.Y.); 3 School of Microelectronics, Northwestern Polytechnical University, Xi’an 710129, China; 4 Department of Physics, Nanchang University, Nanchang 330031, China; 5 Department of Applied Physics, Yale University, New Haven, CT 06511, USA

**Keywords:** silicon nanowire FET, glutamine, pH sensor, potentiometric method, biosensor

## Abstract

In this paper, a potentiometric method is used for monitoring the concentration of glutamine in the bioprocess by employing silicon nanowire biosensors. Just one hydrolyzation reaction was used, which is much more convenient compared with the two-stage reactions in the published papers. For the silicon nanowire biosensor, the Al_2_O_3_ sensing layer provides a highly sensitive to solution-pH, which has near-Nernstian sensitivity. The sensitive region to detect glutamine is from ≤40 μM to 20 mM. The Sigmoidal function was used to model the pH-signal variation versus the glutamine concentration. Compared with the amperometric methods, a consistent result from different devices could be directly obtained. It is a fast and direct method achieved with our real-time setup. Also, it is a label-free method because just the pH variation of the solution is monitored. The obtained results show the feasibility of the potentiometric method for monitoring the glutamine concentrations in fermentation processes. Our approach in this paper can be applied to various analytes.

## 1. Introduction

Glutamine (Gln) is found to be an abundant amino acid in mammalian tissues. In multiple metabolic pathways, it serves as a precursor or product in the central nervous system (CNS), an intermediary in energy metabolism, and a substrate for synthesizing nucleotide bases and glutathione [1,2,3,4]. Also, the catabolism of glutamine is necessary for systemic acid-base balance [5,6]. As a result, the glutamine concentration should be monitored during cell-culture fermentation [7]. 

To detect glutamine, various techniques have been described in the literature. Methods such as high-performance liquid chromatography (HPLC) [8] and gas chromatography-mass spectrometry (GC-MS) [9] are expensive and lab-intensive. For capillary electrophoresis (CE) [10], liquid chromatography-mass spectrometry (LC-MS) [11], and liquid chromatography-tandem mass spectrometry (LC-MS-MS) [12], derivatization is required, which are time-consuming. A much more convenient way is an amperometric method that will employ thin-film electrodes to be a sensor. It will depend on the high consistency of the fabrication process [13]. This is difficult. Also, some methods utilize a labeled substrate. This may affect the interaction between the substrate and the enzyme. To achieve direct, rapid, and label-free detection in biological and chemical applications, much attention has been paid to the nano-scale field-effect transistor (FET)-based devices, such as Si nanowires (SiNWs) [14,15,16,17,18,19,20,21], Si nanoribbons [22,23,24], carbon nanotubes (CNTs) [25], metal oxide nanowires (NWs) [26], metal oxide TFTs [27,28], and graphene [29].

In this paper, we employed the Silicon Nanowire Field Effect Transistor (NWFET) to serve as a biosensor. The variation of the surface potential of the NWFET was recorded, which corresponds to the hydrolyzation of the glutamine. It is a potentiometric method with FET, which means we monitored the potential change caused by the bioprocesses. The Sigmoidal function was employed to describe the relationship between the values of the pH of the solution and the concentration of the substrate, which helped us to quickly determine the density of the glutamine. The NWFET is CMOS-compatible and based on top-down fabrication methods, which means that the device will have high reproducibility for mass production at a low cost [15,17,30,31,32]. Also, they are easily integrated into a detection system. Si nanowire biosensors with a highly sensitive and excellent signal-to-noise ratio (SNR) have been widely reported [19,33,34,35]. Consequently, they are very suitable for monitoring metabolic processes.

## 2. Experiment

### 2.1. Materials

L-glutamine and sodium acetate were purchased from Sigma-Aldrich (St. Louis, MO, USA). The enzyme is L-glutamine amidohydrolase (EC 3.5.1.2) (recombinant, expressed in Escherichia coli), bought from Megazyme (Ireland). It has a specific activity of ~515 U/mg (25 °C, pH 4.9 on L-glutamine). The enzyme is diluted (1:20) in 5 mM sodium acetate buffer with pH 4.9.

### 2.2. Si Nanowire FET Setup and the Biosensing Method

**Figure 1:**
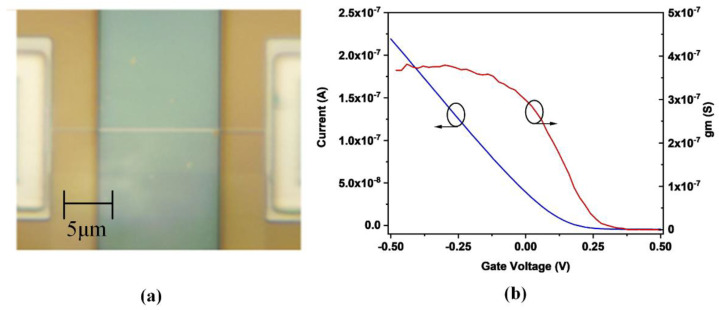
(**a**) Picture of the nanowire FET with a protective SiO_2_ layer. (**b**) transfer current and transconductance of the nanowire FET.

**Figure A1:**
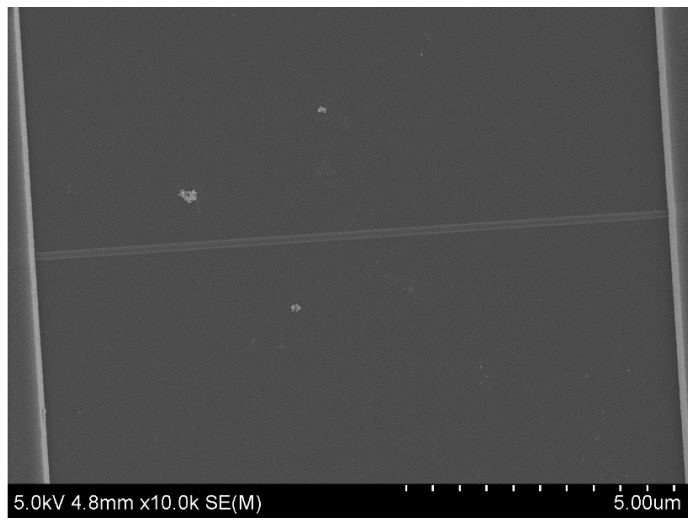
Close-up scanning electron micrograph of a single nanowire post-fabrication.

The nanowire FET used in this paper is based on a “top-down” method as reported in the literature [36]. It is compatible with CMOS processing. The nanowire is shown in Figure 1a. A more legible image of the nanowire is given in Figure A1 in Appendix A. It has an Al_2_O_3_ layer with a typical thickness below 15 nm, which serves as a gate dielectric layer. To isolate the region excepting the Al_2_O_3_ layer from the solution in Figure 1a, a protective SiO_2_ layer was deposited. The p-type SiNW were fabricated on silicon-on-insulator (SOI) wafers. This produces a good performance in gate leakage. To obtain better device uniformity, one nanowire device contained 5 parallel nanowire FETs. The total length of the sensing area of a SiNW is 12 µm.

Figure 1b shows the transfer current and transconductance of the nanowire FET. Based on the characteristic of the nanowire FET, we can choose a suitable quiescent bias to get a large sensitivity region, which should make the range of interest potential in the liner region. Besides, the transconductance of the nanowire FET should also be taken into consideration. The best region should be chosen near the largest transconductance. It is due to that the signal-to-noise ratio is maximized [37].

**Figure 2:**
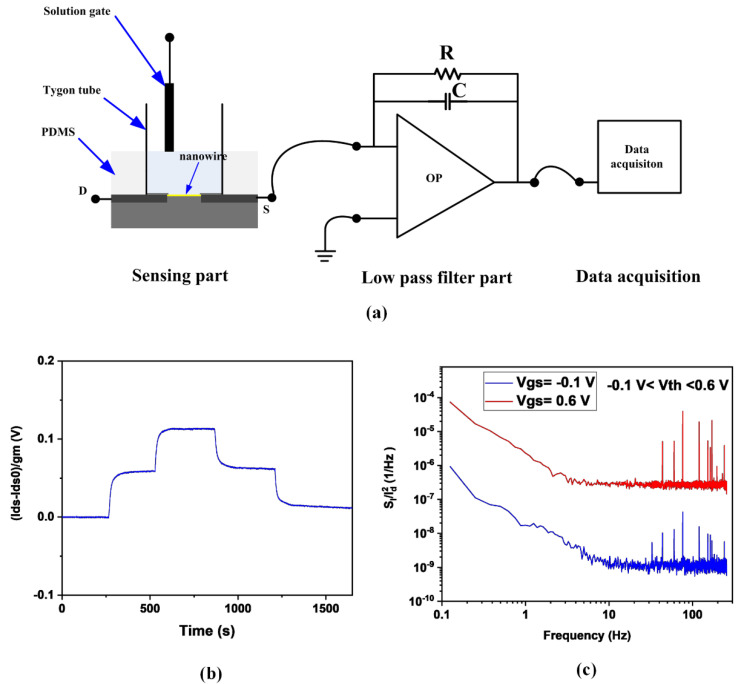
(**a**) Schematic diagram of the multiplexed detection setup. (**b**) pH sensing over the range of interest from a representative device. (**c**) normalized noise current power spectral density of the nanowire FET.

**Figure A2:**
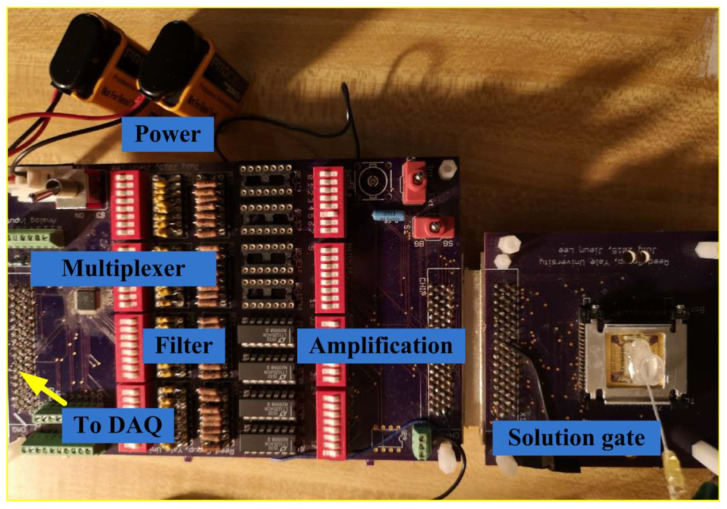
Photograph of nanowire FET measurement setup.

The photograph of the nanowire FET measurement setup is shown in Figure A2 in Appendix A. Its schematic is shown in Figure 2a. It contains three parts: the sensing part, the low pass filter, and the data acquisition part. The reservoir on the SiNWs devices is made with the Tygon tube, which is fixed with epoxy. The Ag/AgCl wires electrode is for the pseudo reference electrode, which is used for applying the quiescent bias. The low pass filter part consists of a quad low noise operational amplifier (The type is LT1125), which is configured as a current-to-voltage converter. The SiNWs device is wire-boned to connect the low pass filter part. The data acquisition employs a USB national instrument Data Acquisition (NI USB DAQ) Card. The custom Labview software is used as an interface for controlling and reading data [38]. For the sensing part, the PDMS was used to reduce the influence of the potential of the bias.

**Figure A3:**
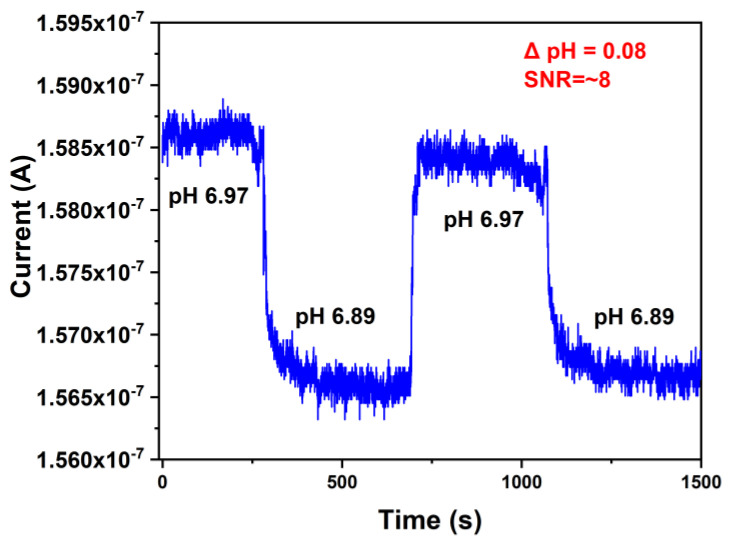
Narrow range pH sensing data from the device, showing the detection of a change in 0.08 pH with a measured signal-to-noise ratio of ~8.

To obtain the device’s sensitivity, the solution with different pH was continuously introduced via a microfluidic channel for the test. Here, buffers of pH 4, 5, and 6 are flowing over the SiNWs to assess the device’s sensitivity to pH, which are interest regions for this experiment. The results are shown in Figure 2b. The Δ*ψ* is in accordance with the ordinate, defined as surface potential. Its values can be acquired by taking the difference between the staring current values and the measured real-time current and dividing by the device transconductance, which has the expression (1). Since the potentiometric biosensor employing the microfluidics and flow system for analytes detection will be influenced by the streaming potential, it is essential to keep the flow-induced noise small. The normalized noise current power spectral density of two representative gate voltages is shown in Figure 2c, which has a smaller value than the data shown in [38]. It indicates that the influence of the streaming potential on the measurement data in Figure 2c is small. Based on the measured results, 16 samples/s were chosen as the sample rate for the measurement.

(1) $$\Delta \psi =\frac{Ids-Ids_{0}}{g_{m}}$$

where the *Ids* is the measured real-time current, *Ids*_0_ is the measured current at the starting time, *g_m_* is the device transconductance. The pH sensitivity of the typical device is obtained as 58.4 mV/pH, which is close to the Nernst limit of 59.1 mV/pH at room temperature. The device has a measured limit of detection (LOD) of 0.01 pH. The narrow range pH sensing data from the device is shown in Figure A3 in Appendix A.

**Figure 3:**
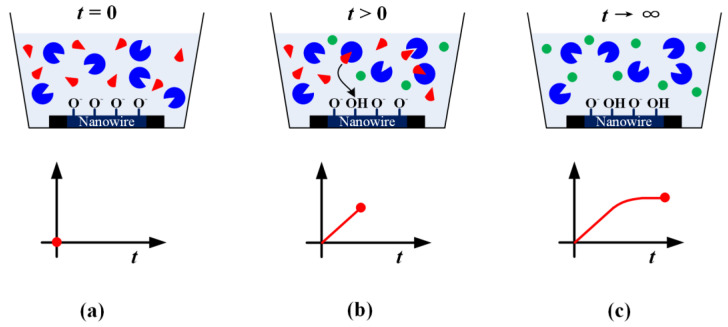
Overview of the biosensing method to detect the substrate (red) by employing the enzyme (blue). (**a**) Beginning of the reaction; (**b**) the substrate is catalyzed by the enzyme to be converted to product (green) with passing time; (**c**) the reaction is finished when all available substrates have been converted to products.

The overview of the proposed biosensing method is shown in Figure 3. The acquired measured data based on the reaction between the enzyme and substrate is detailed. At t = 0, the enzyme (blue) and substrate (red) are put into the reservoir and allowed to react shown in Figure 3a. Figure 3b shows the substrate is catalyzed by the enzyme, and the products (green) have been converted from the substrates. It will change the pH of the solution, and the change in pH can be captured by the nanowire in the solution in real-time, which will induce the current change of the FET. As the reaction in the solution moves on to the end, all the available substrates are catalyzed, hence the signal becomes stable, as shown in Figure 3c. 

### 2.3. The Mechanization of Detection of the Glutamine

Just one hydrolyzation reaction was employed, which is much more convenient compared with the two-stage reactions. The enzyme glutaminase is used in the paper for glutamine metabolism. Glutaminase is a mitochondrial enzyme. It can hydrolyze glutamine to glutamate and ammonia. Humans possess several different glutaminase isoforms. These are encoded by genes on chromosomes two and twelve and the distribution of different forms in human tissues. They have different optimal working conditions [39]. The optimal pH condition for the glutaminase in this paper is 4.9. The hydrolyzation reaction is given below (2):

(2) $${\mathrm{L-Glutamine}+H}_{2}O\overset{\mathrm{L-Glutaminase}}{\to }{\mathrm{L-Glutamate}+\mathrm{NH}}_{4}^{+}$$

**Figure A4:**
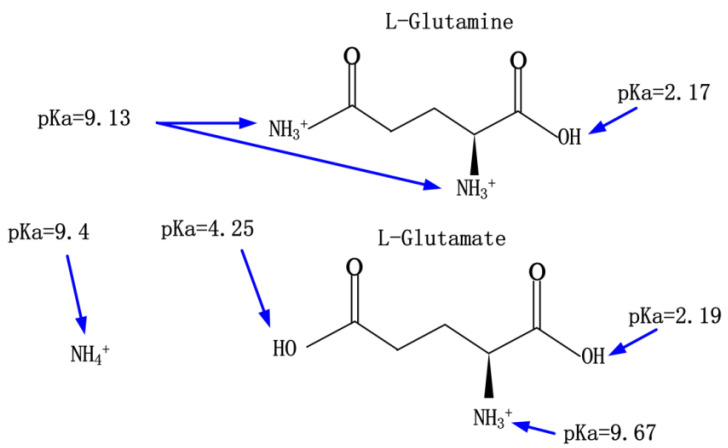
Dissociation constant of different groups in L-glutamine and L-glutamate.

For the hydrolyzation reaction, both the initial buffer with high pH or low pH can be employed to detect the glutamine concentration. Compared to using a solution with a high pH, the experiments are more easily to be a success by using a solution with a low pH. It is due to that sensing layer of Al_2_O_3_ that our devices performed badly in the solution with high pH (pH > 9). So the low pH buffers were chosen to do the experiments. The dissociation constant of different groups in L-glutamine and L-glutamate is shown in Figure A4 in Appendix A. In the hydrolyzation process, new products are produced, which induce the variation of solution pH. To reduce errors, the 40 mM Glutamine solution was prepared. And all other solutions with different concentrations were diluted from it. 

## 3. Results and Discussion

### Measurement

**Figure 4:**
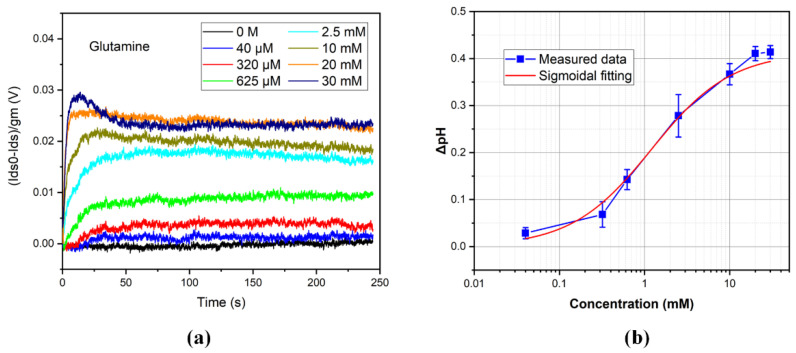
(**a**) Potential signal versus time with different glutamine concentrations. (**b**) pH variation versus glutamine concentration and Sigmoidal fitting result.

The measurements were implemented by using the setup shown in Figure 2a. The surface potential was obtained based on (1). The real-time results are shown in Figure 4a. The procedure is given. (1) put the different concentrations of the glutamine solution into the reservoir shown in Figure 2a (200 μL). (2) start the data acquisition. (3) pipette the enzyme into the reservoir (5 μL, 62.5 U/mL), which corresponds to the case in Figure 3a. The buffers mainly contain L-glutamine, L-glutaminase, and sodium acetate. (4) wait until the real-time curves are stable and stop the data acquisition. At this time, the buffers mainly contain L-glutaminase, sodium acetate, L-glutamate, and ammonia. It may contain L-glutamine when the buffers have a great deal of substrate at step (1).

Based on Figure 4a, the concentration of the glutamine can be determined. However, it isn’t convenient to read the values. The Sigmoidal function is used for modeling in this paper. The equation is given in (3). The fitting values are given in Table A1 in Appendix A. The fitting result is shown in Figure 4b.

(3) $$\Delta pH=\frac{A}{{\left(1+{\left(\frac{x_{0}}{x}\right)}^{h}\right)}^{s}}$$

Figure 4b shows the pH variation corresponding to Figure 4a. The data are extracted from the stable region in Figure 4a. The error bars are also depicted in the figure to show the different device errors. Because the method we used is a potentiometric one, different devices do not have large differences. The relationship between the pH of the solution as a function of the glutamine concentration is proposed by the Sigmoidal function in Figure 4b. The pH of the solution enhanced gradually with the increase of the glutamine concentration. With relatively low concentrations of glutamine, the variation of the solution pH was small. The pH changed a lot when the concentration was in the range of 320 μM and 10 mM, which means that it is an optimal range for detecting glutamine. At a high glutamine concentration, the signal tended toward saturation. It is most likely because of the dramatic change in the solution, which is not suitable for the reaction between the substrate and enzyme. For the whole process, the Sigmoidal function can be successfully used to capture the relationship between them. Glutamine detection can be obtained with a starting concentration of as low as 40 μM, which can be figured out in Figure 4a with the blue line.

## 4. Conclusions

In this paper, we have shown that a fast and direct method can be employed to measure the glutamine-glutaminase interactions by using the silicon nanowire biosensors, which need only one hydrolyzation reaction to determine glutamine concentration. Due to that, the pH variations of the solution can be monitored, and a highly surface-sensitive layer is utilized. This approach is label-free and has a sensitive detection region from ≤ 40 μM to 20 mM for the substrate, respectively. The relationship between the glutamine concentration and pH change of the solution can be depicted by using the Sigmoidal function. Because the surface potentials are recorded, it can effectively improve the inconsistency among different devices compared to the amperometric method. Our approach can be applied to detecting a broad range of substrates.

## Data Availability

All of the data is contained within the article.

## Appendix A

**Table A1:** The fitting values for expression (3).

Fitting Parameter	Values
A	0.41381
x0	1.20574
h	0.92656
s	1

## References

[1] Bak L.K., Schousboe A., Waagepetersen H.S. (2006). The glutamate/GABA-glutamine cycle: Aspects of transport, neurotransmitter homeostasis and ammonia transfer. J. Neurochem..

[2] Albrecht J., Sidoryk-Wegrzynowicz M., Zielińska M., Aschner M. (2010). Roles of glutamine in neurotransmission. Neuron Glia Biol..

[3] Amores-Sanchez M.I., Medina M.A. (1999). Glutamine, as a precursor of glutathione, and oxidative stress. Mol. Genet. Metab..

[4] Coster J., McCauley R., Hall J. (2004). Glutamine: Metabolism and application in nutrition support. Asia Pac. J. Clin. Nutr..

[5] Patience J.F. (1990). A review of the role of acid-base balance in amino acid nutrition. J. Anim. Sci..

[6] Kim M.-H., Kim H. (2017). The Roles of Glutamine in the Intestine and Its Implication in Intestinal Diseases. Int. J. Mol. Sci..

[7] Bäcker M., Delle L., Poghossian A., Biselli M., Zang W., Wagner P., Schöning M. (2011). Electrochemical sensor array for bioprocess monitoring. Electrochim. Acta.

[8] Ecksteina J.A., Ammermanb G.M., Reveles J.M., Ackermanna B.L. (2008). Analysis of glutamine, glutamate, pyroglutamate, and GABA in cerebrospinal fluid using ion pairing HPLC with positive electrospray LC/MS/MS. J. Neurosci. Methods.

[9] Anderson L.W., Zaharevitz D.W., Strong J.M. (1987). Glutamine and glutamate: Automated quantification and isotopic enrichments by gas chromatography/mass spectrometry. Anal. Biochem..

[10] Lee Y.-H., Lin T.-I. (1994). Capillary electrophoretic determination of amino acids with indirect absorbance detection. J. Chromatogr. A.

[11] Fujii K., Ikai Y., Mayumi T., Oka H., Suzuki A.M., Harada K.-I. (1997). A Nonempirical Method Using LC/MS for Determination of the Absolute Configuration of Constituent Amino Acids in a Peptide: Elucidation of Limitations of Marfey’s Method and of Its Separation Mechanism. Anal. Chem..

[12] Qu J., Chen W., Luo G., Wang Y., Xiao S., Lingb Z., Chen G. (2002). Rapid determination of underivatized pyroglutamic acid, glutamic acid, glutamine, and other relevant amino acids in fermentation media by LC-MS-MS. Analyst.

[13] Bäcker M., Rakowski D., Poghossian A., Biselli M., Wagner P., Schöning M. (2013). Chip-based amperometric enzyme sensor system for monitoring of bioprocesses by flow-injection analysis. J. Biotechnol..

[14] Cui Y., Wei Q., Park H., Lieber C.M. (2001). Nanowire Nanosensors for Highly Sensitive and Selective Detection of Biological and Chemical Species. Science.

[15] Stern E., Klemic J.F., Routenberg D.A., Wyrembak P.N., Turner-Evans D.B., Hamilton A.D., LaVan D.A., Fahmy T.M., Reed M.A. (2007). Label-free immunodetection with CMOS-compatible semiconducting nanowires. Nature.

[16] Stern E., Wagner R., Sigworth F.J., Breaker R., Fahmy T.M., Reed M.A. (2007). Importance of the Debye Screening Length on Nanowire Field Effect Transistor Sensors. Nano Lett..

[17] Duan X., Li Y., Rajan N.K., Routenberg D.A., Modis Y., Reed M.A. (2012). Quantification of the affinities and kinetics of protein interactions using silicon nanowire biosensors. Nat. Nanotechnol..

[18] Mu L., Droujinine I.A., Lee J., Wipf M., Davis P., Adams C., Hannant J., Reed M.A. (2017). Nanoelectronic Platform for Ultrasensitive Detection of Protein Biomarkers in Serum using DNA Amplification. Anal. Chem..

[19] Chen K.-I., Li B.-R., Chen Y.-T. (2011). Silicon nanowire field-effect transistor-based biosensors for biomedical diagnosis and cellular recording investigation. Nano Today.

[20] Leonardi A.A., Lo Faro M.J., Di Franco C., Palazzo G., D’Andrea C., Morganti D., Manoli K., Musumeci P., Fazio B., Lanza M. (2020). Silicon nanowire luminescent sensor for cardiovascular risk in saliva. J. Mater. Sci. Mater. Electron..

[21] Leonardi A.A., Faro M.J.L., Irrera A. (2021). Biosensing platforms based on silicon nanostructures: A critical review. Anal. Chim. Acta.

[22] Stern E., Vacic A., Rajan N.K., Criscione J.M., Park J., Ilic B.R., Mooney D., Reed M.A., Fahmy T.M. (2009). Label-free biomarker detection from whole blood. Nat. Nanotechnol..

[23] Mu L., Droujinine I.A., Rajan N.K., Sawtelle S.D., Reed M.A. (2014). Direct, Rapid, and Label-Free Detection of Enzyme–Substrate Interactions in Physiological Buffers Using CMOS-Compatible Nanoribbon Sensors. Nano Lett..

[24] Chen S., Zhang S.-L. (2011). Contacting versus Insulated Gate Electrode for Si Nanoribbon Field-Effect Sensors Operating in Electrolyte. Anal. Chem..

[25] Zhang Y., Arugula M.A., Wales M., Wild J., Simonian A.L. (2015). A novel layer-by-layer assembled multi-enzyme/CNT biosensor for discriminative detection between organophosphorus and non-organophosphrus pesticides. Biosens. Bioelectron..

[26] Zhang R., Curreli M., Thompson M.E. (2011). Selective, Electrochemically Activated Biofunctionalization of In_2_O_3_ Nanowires Using an Air-Stable Surface Modifier. ACS Appl. Mater. Interfaces.

[27] Smith J.T., Shah S.S., Goryll M., Stowell J.R., Allee D.R. (2014). Flexible ISFET Biosensor Using IGZO Metal Oxide TFTs and an ITO Sensing Layer. IEEE Sensors J..

[28] Guo D., Zhuo M., Zhang X., Xu C., Jiang J., Gao F., Wan Q., Li Q., Wang T. (2013). Indium-tin-oxide thin film transistor biosensors for label-free detection of avian influenza virus H5N1. Anal. Chim. Acta.

[29] Suvarnaphaet P., Pechprasarn S. (2017). Graphene-Based Materials for Biosensors: A Review. Sensors.

[30] Kaisti M. (2017). Detection principles of biological and chemical FET sensors. Biosens. Bioelectron..

[31] Shariati M. (2018). The field effect transistor DNA biosensor based on ITO nanowires in label-free hepatitis B virus detecting compatible with CMOS technology. Biosens. Bioelectron..

[32] Tran D.P., Pham T.T.T., Wolfrum B., Offenhäusser A., Thierry B. (2018). CMOS-Compatible Silicon Nanowire Field-Effect Transistor Biosensor: Technology Development toward Commercialization. Materials.

[33] Chua J.H., Chee R.-E., Agarwal A., Wong S.M., Zhang G.-J. (2009). Label-Free Electrical Detection of Cardiac Biomarker with Complementary Metal-Oxide Semiconductor-Compatible Silicon Nanowire Sensor Arrays. Anal. Chem..

[34] Shen M.-Y., Li B.-R., Li Y.-K. (2014). Silicon nanowire field-effect-transistor based biosensors: From sensitive to ultra-sensitive. Biosens. Bioelectron..

[35] Ahmad R., Mahmoudi T., Ahn M.-S., Hahn Y.-B. (2018). Recent advances in nanowires-based field-effect transistors for biological sensor applications. Biosens. Bioelectron..

[36] Knopfmacher O., Tarasov A., Fu W., Wipf M., Niesen B., Calame M., Scho-nenberger C. (2010). Nernstlimitindual-gated Si-nanowire FET sensors. Nano Lett..

[37] Rajan N.K., Routenberg D.A., Reed M.A. (2011). Optimal signal-to-noise ratio for silicon nanowire biochemical sensors. Appl. Phys. Lett..

[38] Lee J., Wipf M., Mu L., Adams C., Hannant J., Reed M. (2017). Metal-coated microfluidic channels: An approach to eliminate streaming potential effects in nanobiosensors. Biosens. Bioelectron..

[39] Brown G., Singer A., Proudfoot M., Skarina T., Kim Y., Chang C., Dementieva I., Kuznetsova E., Gonzalez C.F., Joachimiak A. (2008). Functional and Structural Characterization of Four Glutaminases from Escherichia coli and Bacillus subtilis. Biochemistry.

